# Supplementary material for: DFT Simulation-Based Design of 1T-MoS2 Cathode Hosts for Li-S Batteries and Experimental Evaluation
Source: Int J Mol Sci. 2022 Dec 9;23(24):15608. doi: 10.3390/ijms232415608 (PMC9779699; doi:10.3390/ijms232415608)
Supplement: Supplementary file 1 [file ijms-23-15608-s001.zip › ijms-2053325-supplementary.pdf]

Supporting Information

DFT Simulation-Based Design of 1T-MoS_2 Cathode Hosts for Li-S Batteries and Experimental Evaluation

Elaheh Hojaji [1,2], Eleftherios I. Andritsos [1,2], Zhuangnan Li [3], Manish Chhowalla [3], Constantina Lekakou [1,*] and Qiong Cai [2,*]

[1] Department of Mechanical Engineering Sciences, Faculty of Engineering and Physical Sciences, University of Surrey, Guildford GU2 7XH, UK
[2] Department of Chemical and Process Engineering, Faculty of Engineering and Physical Sciences, University of Surrey, Guildford GU2 7XH, UK
[3] Department of Materials Science and Metallurgy, University of Cambridge, Cambridge CB3 0FS, UK
* Correspondence: c.lekakou@surrey.ac.uk (C.L.); q.cai@surrey.ac.uk (Q.C.)

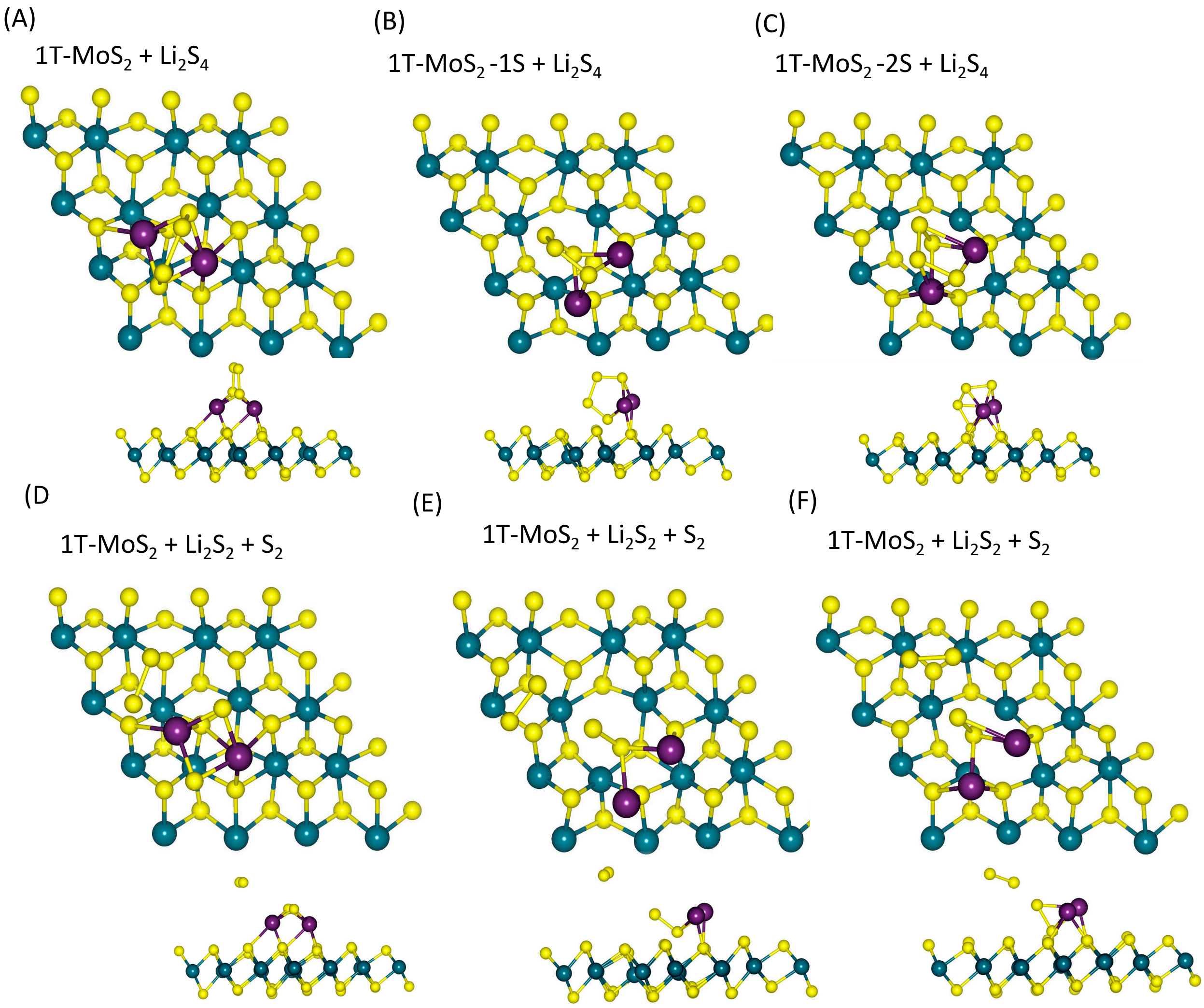

Figure S1. Top views and side views of the optimised geometry of adsorbed Li_2S_4 on (A) 1T-MoS_2, (B) 1T-MoS_2-1S, (C) 1T-MoS_2-2S; (D), (E), (F) its dissociated products on 1T-MoS_2, 1T-MoS_2-1S, 1T-MoS_2-2S, respectively for the reaction pathway of $Li_2S_4 \rightarrow Li_2\ S_2 + S_2$. The S-S bond length was less than 5 Å.

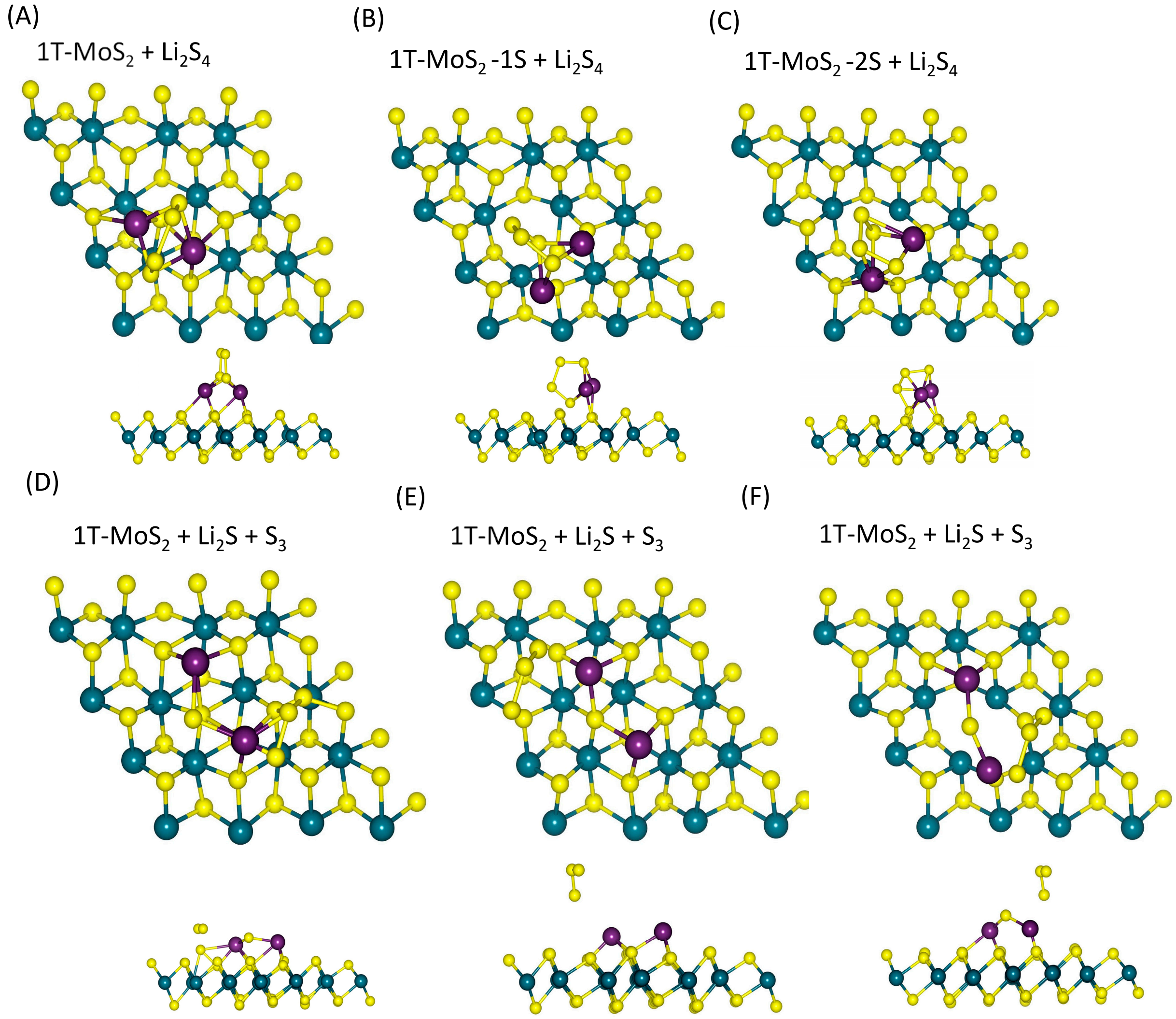

Figure S2. Top views and side views of the optimised geometry of adsorbed Li_2S_4 on (A) 1T-MoS_2, (B) 1T-MoS_2-1S, (C) 1T-MoS_2-2S; (D), (E), (F) its dissociated products on 1T-MoS_2, 1T-MoS_2-1S, 1T-MoS_2-2S, respectively for the reaction pathway of $Li_2S_4 \rightarrow Li_2S + S_3$. The S-S bond length was less than 5 Å.
